# Increases in norepinephrine release and ovarian cyst formation during ageing in the rat

**DOI:** 10.1186/1477-7827-7-64

**Published:** 2009-06-16

**Authors:** Eric Acuña, Romina Fornes, Daniela Fernandois, Maritza P Garrido, Monika Greiner, Hernan E Lara, Alfonso H Paredes

**Affiliations:** 1 Laboratory of Neurobiochemistry, Department of Biochemistry and Molecular Biology, Faculty of Chemistry and Pharmaceutical Sciences, Universidad de Chile, Chile

## Abstract

**Background:**

Depletion of ovarian follicles is associated with the end of reproductive function in ageing females. Recently, it has been described that this process parallels increases in the concentration of norepinephrine (NE) in the rat ovary. In sexually mature rats, experimentally-induced increases in the sympathetic tone of the ovary is causally related to ovarian cyst formation and deranged follicular development. Thus, there is a possibility that increased ovarian NE concentrations represent changes in the activity of sympathetic nerves, which consequently participate in the process of ovarian cyst formation observed during ageing in the human and experimental animal models.

**Methods:**

Sprague-Dawley rats between 6 and 14 months old were used to analyse the capacity of the ovary to release ^3^H-NE recently incorporated under transmural depolarisation in relation to changes in the ovarian follicular population. Morphometric analysis of ovarian follicles and real time PCR for Bcl2 and Bax mRNA were used to assess follicular atresia.

**Results:**

From 8 months old, the induced release of recently incorporated ^3^H-norepinephrine (^3^H-NE) from the ovary and ovarian NE concentrations increased, reaching their peak values at 12 months old and remained elevated up to 14 months old. Increases in sympathetic nerve activity paralleled changes in the follicular population, as well as disappearance of the corpus luteum. In contrast, luteinised follicles, precystic follicles, and cystic follicles increased. During this period, the relationship between Bax and Bcl2 mRNAs (the proapoptotic/antiapoptotic signals) increased, suggesting atresia as the principal mechanism contributing to the decreased follicular population. When NE tone was increased, the mRNA ratio favoured Bcl2 to Bax and antiapoptotic signals dominated this period of development. Thus, these changing ratios could be responsible for the increase in luteinised follicles, as well as precystic and cystic follicles.

**Conclusion:**

These data suggest that the ageing process in the ovary of the Sprague-Dawley rat is accompanied by an increased sympathetic tone of the ovary. Consequently, this sympathetic change could be related to a neuroendocrine-driven formation of a polycystic condition similar to that observed in the sympathetic-activated adult ovary.

## Background

Reproductive senescence in mammals is a poorly understood process. Moreover, understanding the mechanism underlying the loss of ovarian function is especially important because of an increasingly aged population and the fact that pregnancy has been postponed continuously during recent decades [1,2]. Diverse hypotheses for age-related dysfunction have been postulated. The loss of reproductive function with ageing has been correlated with depletion of the oocyte pool (ovarian levels), dysfunction at the central level (hypothalamus), or a combination of both processes [3-6]. In mice, the simple depletion of the ovarian pool of oocytes does not seem to be the primary factor involved in reproductive failure associated with age, which is supported by reports of a substantial oocyte pool remaining upon cycle termination [7]. Female rats have an expected life span of 2–3 years, but exhibit an increasing frequency of irregular reproductive cycles during middle age (8–12 months) and become acyclic when they are old (17–21 months) [8,9]. It has recently been determined that ageing in rats is accompanied by increasing NE levels in the ovary [10]. Thus, ovarian NE could be involved in the loss of reproductive performance found since middle age. It has been reported previously [11-14] that an experimentally-induced increase in ovarian sympathetic tone in the adult rat is causally involved in the transformation of preovulatory follicles (through a chain of neurotrophic-dependent mechanisms) to a polycystic ovary condition (PCO) with a simultaneous loss of estrual cycling.

There is little information regarding the role of sympathetic activation within the ovary of middle aged animals and the potential involvement in the loss of reproductive function. If the recently reported increase in NE concentration [10] is related to increased nerve activity and not to an accumulation of NE during ageing in the ovary, it is probably responsible for the changes in follicular development accompanying the age related decreases in ovarian function. Since some studies have found decreased and others increased levels of NE during ageing [10,15], the present work sought to correlate changes in NE concentration and the release capacity of the nerves with the dynamic changes in the ovary follicles and reproductive performance. This study could be especially important considering that once a PCO is established in humans, the condition does not disappear until senescence and it can even continue after menopause [16].

## Methods

### Animals

Adult female Sprague-Dawley rats derived from a stock kept at the University of Chile were used. Rats from 6 to 14 months old were maintained in individual cages at 20–23°C in an alternating 12 h light:dark regimen (lights off from 1900 h to 0700 h) with food and water ad libitum. Forty-five rats were used, which were divided into the following five age groups with 9 rats per group: 6, 8, 10, 12 and 14 months old. Four rats per group were used to examine ^3^H-NE release and for morphology experiments (one ovary for each technique either from the left or right side). The other 5 rats per group were used to measure endogenous ovary NE content, as well as for RNA extraction and real-time PCR (one ovary for each technique either from the left or right side). Table 1 shows the percentage of dams that became pregnant between 4 and 10 months old and the number of live pups born. Data were obtained from the animal facility for the Faculty of Chemistry and Pharmaceutical Sciences, Universidad de Chile. The groups were analysed in a retrospective form. At 6 months old, female rats exhibited a decrease in the number of pregnancies and delivered fewer pups. These data are well correlated with the decrease in the frequency of normal estrous cycles found in 10 month old rats. Interestingly, the 14 month old rats presented as two groups with irregular cycling profiles. Namely, one group (about 50% of rats studied at this age) presented with a continuous estrous condition. In contrast, the other 14 month old rats presented with elongated transitions between proestrus and diestrus, thereby suggesting a non-ovulatory condition.

**Table 1:** Changes in pregnancy rate during ageing

Age rats (months)	4	5	6	7	8	9	10
Pregnancies (%)	69.8	92.1	85.7	69.8	50.8	22.2	4.8
Number of pups born live	8.3 ± 0.6	10.7 ± 0.42	9.7 ± 0.43	6.8 ± 0.53	4.9 ± 0.87	2.6 ± 0.63	0.5 ± 1.89

Shown is the percentage of rats that became pregnant at different ages and the number of live pups born from rats between 4 to 10 months old. Data were obtained from the animal facility of the Faculty of Chemistry and Pharmaceutical Sciences, Universidad de Chile. Each point represents means ± SEM of 63 rats.

All animal procedures were performed following protocols previously approved by the Institutional Ethic Committee of the Faculty of Chemical and Pharmaceutical Sciences, Universidad de Chile, and experiments were conducted in accordance with the International Guiding Principles for Biomedical Research Involving Animals as promulgated by the Society for the Study of Reproduction. The animals were sacrificed by decapitation, trunk blood was collected, and serum was stored at 4°C in order to determinate progesterone, androstenedione, and estradiol levels. The ovaries were collected and immediately frozen at -80°C, with the exception of the four left-sided ovaries in each group that were fixed for morphological studies. In each rat, the stage of the estrual cycle was determined by microscopic analysis of the predominant cell type obtained from daily (Monday through Friday) vaginal smears. All rats were sacrificed during diestrus.

### Ovarian morphology

Fresh ovaries were immersed in Zamboni fixative, embedded in paraffin, cut into 6 um sections, and stained with hematoxylin and eosin. The presence of preantral, antral, atretic, precystic (type 3), and cystic follicles was analysed according to Lara et al. [13]. All slices were analysed and preantral follicles corresponded to those with no antral cavity, i.e. primordial, primary and secondary follicles. Antral follicles presented with an antral cavity (antrum), i.e. tertiary and preovulatory follicles. Total preantral follicles corresponded to healthy and atretic follicles. The same procedure was followed to measure antral follicles. With the exception of primordial follicles (presenting flat cells around), preantral follicles were defined as follicles without any antral cavity and with one or more layers of granulosa cells. Atretic follicles were defined as those follicles with more than 5% of cells that had pyknotic nuclei in the largest cross-section and showed shrinkage and an occasional breakdown of the germinal vesicle. Antral follicles were counted when the nucleus of the oocyte was visualised. Cystic follicles were defined as those follicles devoid of oocytes and displaying a large antral cavity, an enlarged thecal cell layer, and a thin (mostly monolayer) granulosa-cell compartment that contained apparently healthy cells. Type 3 follicles were defined according to the criteria proposed by Brawer and colleagues [17,18]. These follicles were large, devoid of oocytes, and contained four or five plicated layers of small, densely packed granulosa cells surrounding a very large antrum with a seemingly normal thecal compartment. Type 3 follicles may represent precystic follicular structures [17,18]. Our previous observations suggested the existence of a transitional stage between healthy preovulatory follicles and the type 3 follicles previously described [13]. Luteinised follicles are characterised by the presence of luteinised granulosa cells (a big cytoplasm similar to luteal cells), and have an antral cavity (although there is not an oocyte) [19].

### Estradiol, androstenedione and progesterone assays

Serum estradiol, androstenedione, and progesterone levels were measured by an enzyme immunoassay (EIA) following the manufacturer's instructions (Alpco Diagnostics, Windham, New Hampshire, USA). Estradiol sensitivity was 10 ng/ml, and the intra- and inter-assay variations were less than 9.3% and 9.9%, respectively. Progesterone sensitivity was 0.1 ng/ml, and intra- and inter-assay variations were less than 10.6% and 12.6%, respectively. Androstenedone sensitivity was 0.05 ng/ml, and the intra- and inter-assay variations were less than 6.7% and 14.6%, respectively.

### Determination of total adrenal catecholamines

The adrenal glands were weighed and homogenised in 500 μL of ice cold 0.2 N PCA. The homogenate was centrifuged at 12,000 g for 10 min at 4°C. The supernatants were used for colourimetric determination of total catecholamines. As previously reported [20], this method measures NE and epinephrine as a whole by the formation of noradrenochrome and adrenochrome (excitation wavelength of 540 nm) when the sample is oxidised with iodine at a pH higher than 6.0

### Measurement of endogenous tissue NE content

One frozen ovary from each rat was homogenised in 200 μl 0.1 M acetic acid. Subsequently, 50 μl of the homogenate was precipitated with 180 μl 0.2 M HClO_4 _and centrifuged (15,000 g for 15 min). NE was measured in the supernatant by HPLC coupled with electrochemical detection [21]. A 150 μl sample was mixed with 50 mg activated alumina in 1 ml TrisCl 1.5 M, pH 8.3–8.5, containing 2% EDTA. Dihydroxybenzyl amine (DHBA, 2000 pg in 20 μl; Sigma, St. Louis, Missouri, USA) was added as an internal standard. The alumina was rinsed thoroughly with nanopure water and NE was eluted with 100 μl of 0.2 N perchloric acid and centrifuged. Afterwards, 20 μl of the resulting 8 supernatants were injected into a Waters HPLC system equipped with a C18 reverse phase column (Lichrosphere, 60 RP-Select B, Merck, Darmstadt, FR Germany) and an electrochemical detector (Waters 464). The mobile phase contained 0.1 M NaH_2_PO_4_, 0.42 mM octyl-sulphate, 0.02% EDTA and 1.5% acetonitrile (pH 2.5) with a 0.9 ml/min flow rate. The potential of the amperometric detector was set to 0.7 V. Under these experimental conditions, the retention time was 4 min for NE and 10 min for DHBA.

### Uptake and release of NE

The procedure was performed as previously described [22,23], with some modifications. Rats were sacrificed at 6, 8, 12 and 14 months old. The ovaries were rapidly removed through an abdominal mid-line incision. One ovary was frozen at -80°C to isolate total RNA and the other was preincubated for 20 min in Krebs Ringer buffer (KRB) (pH 7.4), gassed with 95%O_2_-5%CO_2_, and then incubated for 30 min at 37°C with 2 μCi ^3^H-NE (New England Nuclear Life Science Products, Boston, Massachuesetts, USA). After washing the tissue in KRB (6 washes of 10 min each) to remove non-incorporated radioactivity, the ovaries were transferred to a thermoregulated superfusion chamber and perifused at a 1.5 ml/min flow rate for 10 min with KRB plus 10 mM tetraethylammonium, a potassium channel blocker which enhances the release of NE from nerve terminals in response to electrical stimulation [24,25]. One-minute fractions were collected. After 3 min, the ovary was subjected to a train of monophasic electrical pulses (80 V, 10 Hz, 10 msec/pulse for 1 min), delivered through a parallel set of platinum electrodes that was generated by a Grass S-4 stimulator (Grass Instruments, Quincy, Massachuesetts, USA). One-minute fractions were collected for an additional 5 min. In each experiment, ovaries from the different age groups were simultaneously stimulated in parallel superfusion chambers. At the end of the experiment, the ovaries were homogenised in 0.4 M perchloric acid, and ^3^H-catecholamine remaining in the tissue was determined by scintillation counting in a Tri-Carb Liquid Scintillation Analyzer 1600TR (Packard Instruments, Meriden, Connecticut, USA), with 72.5% efficiency for ^3^H. These values were used to calculate the radioactivity remaining in the tissue after the experiment. In order to calculate the amount of NE released, the radioactivity present in each of the one-minute samples was determined in the same way. The release, which represented ^3^H-NE overflow from the ovaries, was then expressed as fractional release, i.e. as a percentage of the total radioactivity present in the tissue. The total amount of neurotransmitter released was calculated as the area under the curve after stimulation minus that for basal efflux [22,24].

### Reverse transcriptase reaction

Total RNA was extracted as described by Chomczynski and Sacchi [26] from whole ovaries. Total RNA (5 μg) was subjected to reverse transcription at 42°C for 50 min, using 1.6 mM dNTPs, 10 mM DTT, 176 nM random hexamers (Invitrogen, Carlsbad, California, USA), 25 U RNaseOUT (Invitrogen, Carlsbad, California, USA), 125 U reverse transcriptase SuperScript II (Invitrogen, Carlsbad, California, USA), and first strand buffer in a final volume of 30 μl. The reaction was terminated by heating the samples at 75°C for 10 min.

### Real time PCR

For specific gene amplification, a standard protocol of 40 cycles was used in a MJ Research PT-200 (MJ Research Inc., Watertown, Massachusetts, USA.). A standard real-time PCR reaction mix was prepared containing the following components: 15 μl of Platinum SYBR Green PCR Super MIX UDG (Invitrogen, Carlsbad, CA), 2.4 mM MgCl_2_, 0.16 μM each primer and 2 μl cDNA. After initial polymerase activation at 95°C for 10 min, primer-specific amplification and quantification cycles were run at 60°C for 15 s and 72°C for 20 s. The fluorescence intensity of the double-strand specific SYBR-Green I, reflecting the amount of PCR-product actually formed was read at the end of each elongation step, after previous melting curve analyses to determine the melting points of the PCR products. Then, the amounts of specific initial template mRNA were calculated by determining the time point at which the linear increase of sample PCR product started relative to the corresponding points of a standard curve, which was obtained by serial dilutions of known copy numbers of the corresponding control tissue. To evaluate specific amplification, a final melting curve was created (72–95°C) under continuous fluorescent measurement.

The primers used for amplification were a forward primer (5-CAC-CCT-GGC-ATC-TTC-TCC-TT-3) and reverse primer (5-ACA-CAT-GAC-CCC-ACC-GAA-CTC-3) corresponding to *Bcl2 *mRNA (Genebank access NM_016993). The *Bax *primers were composed of a forward primer (5-AGG-CGA-ATT-GGC-GAT-GAA-CTG-3) and reverse primer (5-TTC-TGA-TCA-GCT-CGG-GCA-CTT-TAG-3) (Genebank access U49729). These primers were from Invitrogen/Life Technologies, Inc. To normalise the quantification of *Bcl2 *and *Bax *mRNAs, ribosomal *18s *mRNA was measured in each protocol. Amounts of *18s *mRNA were determined using a commercially available RT primer pair (Ambion, Austin, Texas, USA). Amplification of *18s *mRNA was performed in a different tube to avoid interference with the amplification of the mRNAs. Reaction tubes lacking RT were used as PCR controls. The RT-PCR products were separated on 2.0% agarose gels, stained with ethidium bromide, and photographed digitally.

### Statistical analysis

Differences between the different ages groups were analysed by one-way ANOVA, followed by the Student-Newman-Keuls multiple comparison test for unequal replication. The level of significance was set to P < 0.05.

## Results

### Changes in the estrual cycling activity and reproductive performance during ageing in rat

**Figure 1:**
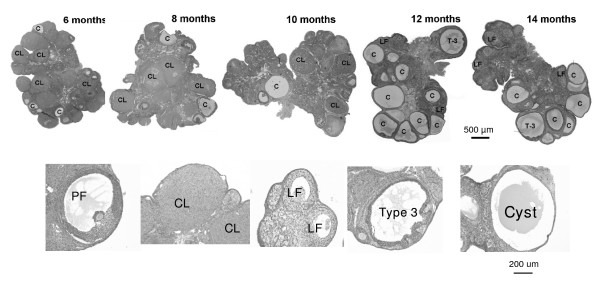
**Morphological aspect of ovaries from adult rats at different age intervals**. Upper panel shows a picture of the ovaries from rats at 6, 8, 10, 12 and 14 months old. CL, Corpus luteum; LF, luteinised follicles, F, antral follicle; C, cyst; Ty-3F, type-3 follicle. Bar, 500 um. Lower panel shows the morphological aspect of a normal preovulatory follicle (PF), corpus luteum (CL), a luteinised follicle (LF), a type 3 follicle and a follicular cyst (C). Bar: 200 um.

The morphological analysis of the ovary (Figure 1) showed important changes starting from 10 months old. These changes were characterised by a gradual disappearance of the corpus luteum, the appearance of luteinised follicles, and of cystic-like follicular structures.

### Changes in follicular dynamics during ageing in the rat

**Figure 2:**
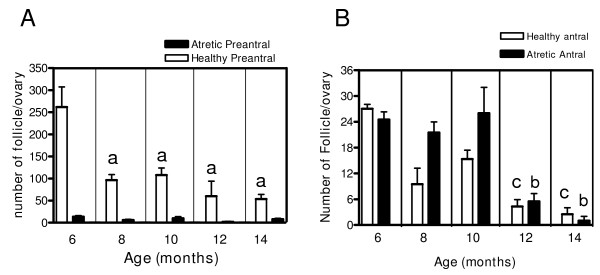
**Changes in preantral and antral follicle population during ageing in the rat**. The figure represents follicular quantification. Panel A corresponds to the total number of healthy and atretic preantral follicles and panel B corresponds to healthy and atretic antral follicles according to age. Results are mean value ± SEM of n = 4 experiments. a = p < 0.05 vs 6 months old; b = p < 0.05 vs 6, 8, 10 months old; c = p < 0.05 vs. 6, 10 months old.

As shown in Figure 2, from the 6 to 8 month transition, there is an important decrease in the number of healthy preantral follicles (Figure 2A). After this age, there was a decrease and it remained low up to 14 months old. Atresia numbers were maintained at low levels during the entire study period. The antral population (Figure 2B), showed a decrease from 6 to 14 months old with higher levels of atresia up to 10 months old. At 14 months old, a small number of healthy antral follicles were found (two or three per ovary). Because atresia was increased up to 10 months and decreased after this period, the atretic to healthy ratio initially presented as a period (up to 10 months old) in which there was a sharp increase in the ratio that decreased after this age.

### Changes in hormone plasma levels in relation to follicular structures in the ovary

**Figure 3:**
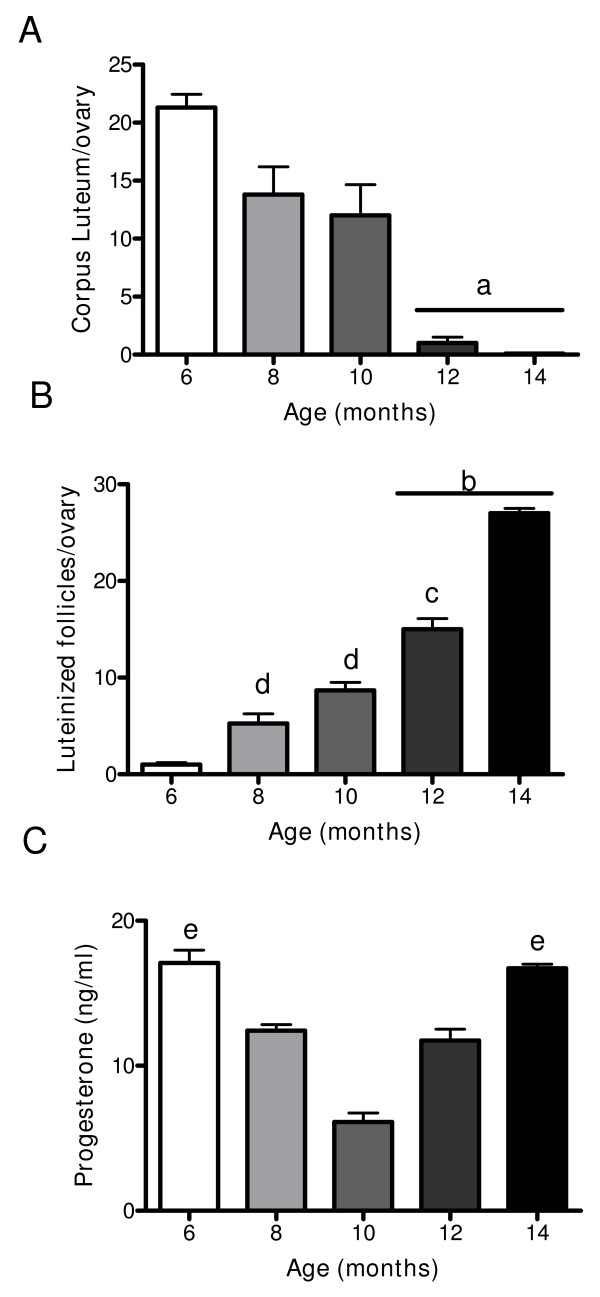
**Changes in corpus luteum, luteinized follicles in the ovary and in progesterone during aging in the rat**. (A) represents number of corpus luteum, (B) represents number of luteinized follicles in the ovary and quantified as it was described in methodology section and (C) serum level of progesterone during aging in the rat measured by EIA. Results are mean value ± SEM of n = 4 experiments. a = p < 0.001 vs. 6, 8, 10 months, b = p < 0.001 vs. all groups; c = p < 0.01 vs. 8 months; c = p < 0.05 vs 6 months; e = p < 0.01 vs 10 months.

**Figure 4:**
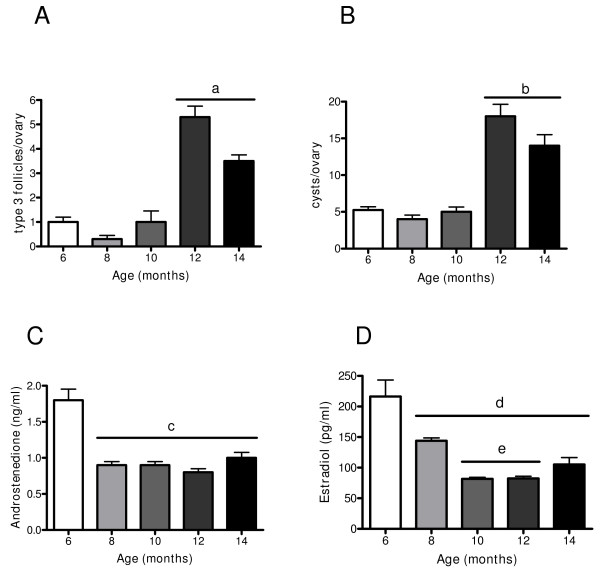
**Changes in the number of preovulatory follicles, type-3 follicles, and cystic follicles in the ovary and in androstenedione and estradiol serum levels during aging in the rat**. (A) represents number of type-3 follicles, (B) represents number of cystic follicles in ovary rat quantified like was described in methodology section and (C) androstenedione serum level and (D) estradiol serum levels during aging in the rat measured by EIA. Results are mean value ± SEM of n = 4 experiments. a = p < 0.05 vs 6, 8 and 10 months; b = p < 0.01 vs 6 and 8 month; c = p < 0.01 vs 6 months; d = p < 0.01 vs 6 months; e = p < 0.05 vs 8 months.

As shown in Figure 1, no corpus luteum appeared in the 10 month old rats. The morphometric analysis confirmed this observation (Figure 3A) and only a few corpus lutei were found from 12 month old rats. However, there was a continuous increase in the number of luteinised follicles with age (Figure 3B), which was evident from 10 month old rats. When plasma progesterone levels were measured, a decrease from 6 to 10 months old was found, but an increase in the plasma levels of the hormone was also found from this age up to 14 months old (Figure 3C). Although a decrease in the preovulatory pool of follicles with age was found, a concomitant increase in two anomalous follicular structures (type-3 follicles and follicular cysts) was also found from 10 months old (Figure 4A and 4B). In adult rats, it has previously been described that type-3 follicles represent a previous stage in follicular cyst formation [13]. Androstenedione and estradiol plasma levels (Figure 4C and 4D) exhibit an initial decrease from 6 to 8 months old and were maintained at the same levels up to the last age studied (14 months old).

### Changes in plasma norepinephrine and in the concentration of catecholamines in the adrenal gland, celiac ganglia, and ovary during ageing

As shown in Table 2, NE plasma levels have a tendency to decrease with age with the exception of the 14 month old group. The decrease in NE was also found in the celiac ganglia, but not in the adrenal gland. Regarding the ovary, there was no change in the weight of the ovary during ageing. The total amount of NE in the celiac ganglia was maintained at a high level up to 10 months old when it decreased to 60% of the NE content found in 6 month old rats, and these reduced levels remained unchanged until the end of the study period. In the same way, the ovarian concentration (ng NE/mg of tissue) increased from 8 months to reach higher levels of NE up to 12 months old and remained elevated from 6 to 14 months old (Table 1). No changes in total catecholamines of the adrenal gland were found during any period of the study (Table 2).

**Table 2:** Changes in NE concentrations during rats' ageing

Age:	6 month	8 month	10 month	12 month	14 month
Ovary weight (mg tissue/pair ovaries)	24.13 ± 3.0	23.11 ± 1.6	28.25 ± 3.7	27.57 ± 3.2	25.78 ± 2.6
Ovary (pg NE/mg)	157 ± 48	215 ± 27,5	231 ± 35^a^	375 ± 65.9^b^	216 ± 20.2
Plasma (pg NE/ml)	52.65 ± 9	48.9 ± 9	40.78 ± 9	35.95 ± 10	47.1 ± 11
Celiac Ganglion (ng NE/ganglia)	69.79 ± 8	55.5 ± 10.1	60 ± 14	22.8 ± 8.9^c^	30.2 ± 10^c^
Adrenal (μg catecholamines/mg)	2.14 ± 0.25	2.80 ± 0.6	3.82 ± 0.32	2.98 ± 0.21	3.7 ± 0.33

Results are mean value ± SEM of n = 5 experiments in each age. (a) p < 0.05 vs 6, 8 and 14 months old, (b) p < 0.05 vs all groups studied, (c) p < 0.01 vs 6, 8 and 10 months old.

### Changes in the release of recently incorporated norepinephrine from the ageing ovary

**Figure 5:**
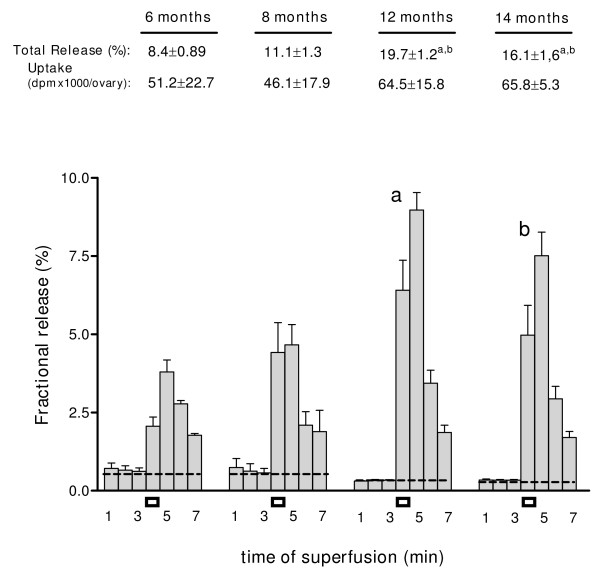
**Ovarian release of ^3^H-NE during ageing in the rat**. Release was induced by electric-induced depolarisation (black rectangles). Results are expressed as percent of ^3^H-NE retained in the tissue at each interval studied. Numbers below the age of rats represent total ^3^H-NE released by electric depolarisation and uptake of NE dpm x1000/ovary. Each bar represents the mean ± SEM of 4 experiments, for each of the ages. a = p < 0.01 vs 6 and 8 months old; b = p < 0.05 vs 6 and 8 months old.

In order to discriminate if the increased concentration of NE represented increased activity of the nerves or accumulation of NE, the release activity of the nerves when the ovary was preloaded with ^3^H-NE was measured. The results demonstrated a constant increase in the stimulation-induced release capacity of the ovary from 8 months old, reaching a peak at 12 months (Figure 5). A significant increase in the area under depolarisation was found at 12 months old and it was maintained at 14 months old. No changes in the uptake capacity of ^3^H-NE was found (legend in Figure 5).

### Changes in pro and antiapoptotic signalling in the ageing ovary

**Figure 6:**
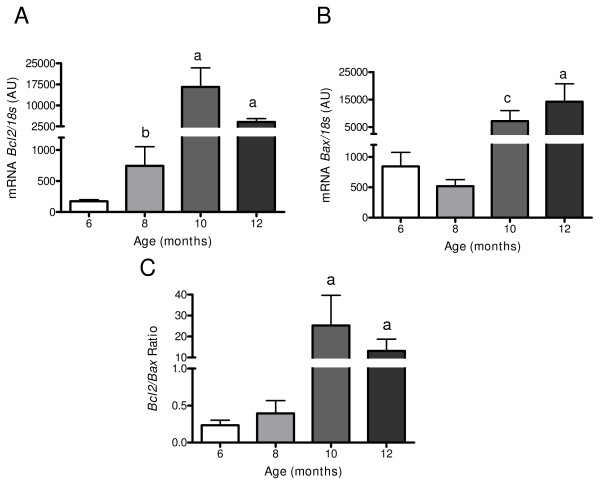
**Changes in Bcl2 and Bax mRNA during aging in the rat**. It is shown Bcl2 (Figure 6A), Bax (Figure 6B) and the ratio in both mRNA (Figure 6C), in the ovary during aging in the rat. Specific mRNA was measured by real time PCR as it was described en methods and normalized by the housekeeping gene ribosomal 18s mRNA. Results are mean value ± SEM of n = 5 experiments. a = p < 0.001 vs 6 and 8 months old; b = p < 0.05 vs 6 months old; c = p < 0.01 vs 6 months old.

After 10 months old, there was a sharp decrease in corpus lutei numbers and in the number of preovulatory follicles suggesting a decreased ovulatory process and probably other developmental pathway changes for preovulatory follicles. Therefore, the mRNA for the proapoptotic protein BAX and the antiapoptotic protein BCL2 were measured (Figure 6). There was an increase in the mRNA for the proapoptotic protein BAX until 10 months old that remained high at older ages. The highest increase of BCL2 was from 10 to 14 months old. Thus, the ratio of *Bcl2 *to *Bax *mRNA increased at 10 and 12 months old.

## Discussion

In the present paper, it was found that the ageing of ovarian function in Sprague-Dawley rats is accompanied by a spontaneous increase in the capacity of the sympathetic nerves of the ovary to release NE and to develop a polycystic condition. We used Sprague-Dawley rats because female rats have an expected life span of 2–3 years, thereby allowing an easier assessment of reproductive function in a relatively short period of time. In addition, almost all of the central and peripheral mechanisms involved in reproductive function either in males or females [27] have been initially described in rats and have been confirmed to occur in humans, thereby justifying its use as a model to understand reproductive function.

### Changes in follicular dynamics with ageing in the Sprague-Dawley rat

Consistent with what has been observed in the reproductive cycles of perimenopausal women [28,29], it was found that reproductive ageing in the Sprague-Dawley rat was characterised by a gradual shift from the normal 4–5 day estrous cycle to cycles of variable length. This result has been previously found by others [30,31], and is characterised by an increasing proportion of nonovulatory cycles, and reduced numbers of developing ovarian follicles, which compromise fertility [32-34]. The age-related change in rodent estrous cyclicity seems to be attributed to the failure to continue producing progesterone in a cyclic fashion and, as it was found in the present study, a constant decrease in plasma progesterone up to 10 months old and older. Cessation of estrual cycling activity was correlated with an important decrease in the number of antral preovulatory follicles, suggesting that the ageing process is a consequence of the disappearance of the preovulatory follicle pool in the ovary. The constant decrease in the total follicular pool of the ovary was probably due to several reasons. First, a sharp decrease was found in the preantral follicle population during the 6 to 8 months old transition period. Second, the increased atresia of antral follicles reached their highest levels when rats were 10 months old. The correlation found between the morphological evidence of atresia, the increased ratio between atretic and healthy antral follicles, and the biochemical marker *Bax*, which was high compared to the mRNA for the antiapoptotic protein BCL2 until 10 months old, indicated that apoptosis predominates in ovarian follicles during the subfertile period [35]. Interestingly, when the cessation of the fertility process started the relationship between the antiapoptotic and apoptotic proteins was reversed. This indicates that when the fertile period ceased (indicated by no estrous cycling activity, no ovulation, and disappearance of CL), the ovary developed mechanisms to increase survival of the remaining follicles. As a result, it presumably developed the new population of luteinised follicles. These luteinised follicles were derived from preovulatory follicles that were ordinarily not going to undergo ovulation. This finding suggested that even though ovulatory cycles had ended, the remaining preovulatory follicles did not go to atresia and they were induced to luteinisation or to become other non-atretic structures in the ovary such as type-3 and cystic follicles. Because of animal age and due to the loss of the capacity to maintain the estrous cycling activity, there is probably no LH preovulatory surge. Consequently, there are constant levels of LH and loss of the pulsatile secretory activity from the hypothalamus [36]. Chavez-Genaro et al [10] recently described that when old rats (in the subfertile age period) are treated with gonadotropin, they are able to ovulate, suggesting a decrease in hypothalamic GnRH control to the hypophysis [37]. Supporting a loss of pulsatile release of gonadotropin as a key factor in developing the morphological changes in the ovary, Risma et al [38] found that the ovaries of mice over-expressing LH were influenced by the constantly high LH levels that provoked strong changes in follicular development, stimulated the formation of luteinised follicles, and induced the appearance of type 3 and cystic follicles. If this were the case for the ageing ovary of the Sprague-Dawley rat, it could be the reason why there were a high number of precystic type-3 follicles and cystic follicles. Almost identical morphological changes in the ovary were described previously by Brawer et al [39] and were repeated in this research [12] by administrating estradiol valerate to sexually mature adult rats. Interestingly, this treatment demonstrated that there was a decrease (and not an increase as in LH-overexpressing mice) in LH levels, and most importantly; there was a loss of pulsatile activity. Thus, the changes in follicular dynamics could be independent of central mechanisms involving local regulators (probably intraovarian), which can then be stimulated by gonadotropins to provoke the loss of the preovulatory follicular pool. Sympathetic nerves are the target tissues for gonadotropins [24] and can be locally stimulated to increase their activity and modify follicular development.

### Sympathetic nerve activity and development of a polycystic condition

It was previously described that a sexually mature adult rat treated with estradiol valerate (EV) developed a similar morphological change in the ovary similar to what we have described in the ovaries of ageing rats. These morphological changes were chronologically related with a previous increase in nerve activity (as measured by NE concentration), and with the capacity of the ovary to release recently incorporated ^3^H-NE after a depolarising stimulus [12,13]. All of these changes were experimentally demonstrated to occur in the ageing rats. The fact that NE release capacity increased during ageing suggests a direct relationship between nerve activity and precystic and cystic structures in the ovary. According to the recent study of Chavez-Genaro [10], peripheral sympathetic denervation by treatment with guanethidine to decrease the NE in the ovary during the subfertile period did not provoke an increase in the number of ova shed in 12 month old denervated rats after gonadotropin stimulation. This finding suggests that if sympathetic nerves participate in the control of ovulatory function at the stage of preovulatory rupture of the follicle or in the recruitment of the follicular population, sympathetic tone may also participate in other steps of follicular development such as when the ageing ovary is marked by decreases in primordial, secondary, and preovulatory follicles. In this regard, sympathetic nerve activity has causally been related to a neurotrophin-induced increase in the activity of ovarian nerves [11,13,40], which stimulate the transition between preovulatory to cystic follicles. Thus, the lack of NE (denervation) probably decreased the number of preovulatory follicles going to type 3 and follicular cysts, and rescued them towards ovulation. Convery et al [18] demonstrated that hemiovarectomy of EV-induced polycystic ovary in rats, leads to ovulation of unhealthy (probably type-3) follicles. If this were the case for the ageing ovary, it could suggest that the increased sympathetic activity found in the ovary induced type 3 and cyst formation because atresia (as assessed by morphological and molecular approaches) decreased at this age. Considering that NE concentrations in the ovary of the rats used in this study increased until 12 months old and were maintained at this elevated level, the increase in antiapoptotic signal and NE activity could be parallel events. Currently, experiments are in progress to test for a causal relationship between increased NE activity and atresia. The fact that the concentration of NE in the celiac ganglia (where sympathetic neurons innervating the endocrine compartment of the ovary originate) decreased at an early age (similar to the plasma levels), strongly suggests that the decrease in the sympathetic nerve supply to the ovary with ageing is a consequence of a more generalised ageing process. It also suggests that the increased activity of the intraovarian compartment of the ovary could be attributed to the increase in local intraovarian signals in which nerve growth factor and its receptors could participate. This phenomena was previously demonstrated for cyst formation in sexually mature rats stimulated with EV [13,40], despite the lack of change found in other tissues innervated by the celiac ganglia such as the adrenal gland.

Supporting this assertion, it has been demonstrated [41] that ovaries from postmenopausal women possess a higher density of sympathetic nerves and ovarian cystic conditions remain in women after menopause [16]. Currently, it is difficult to support a direct relationship between sympathetic tone and ageing ovary function. Nevertheless, this is an important relationship; especially now that people are exposed to environmental factors which may modify their progression towards the end of the reproductive period, as we have demonstrated to occur in rats during early exposure to estradiol [21]. In addition, this is important because women have postponed pregnancies to later in their fertile period due increasing social and/or economic demands. More studies in animal models will provide addition support clarifying the role of sympathetic nerve participation in ovary function during ageing.

## Conclusion

Altogether, these data suggest that an increased intraovarian sympathetic tone participates in the processes that induce the polycystic condition associated with ageing in the Sprague-Dawley rat, and it has opened a new mechanism to control follicular development during natural ageing. The use of rats as a model to investigate the natural ageing of ovary function appears to be a useful model for studying the processes and mechanisms which control follicular development.

## Competing interests

The authors declare that they have no competing interests.

## Authors' contributions

EA: quantification of catecholamines in adrenal gland and NE in celiac ganglion and analysed of reproductive parameters of the rats RF: chromatographic determination of adrenalin in plasma and ovary. DF: primer design and determination of Bcl2 mRNA by RT-Real Time PCR. MPG: primer design and determination of Bax mRNA by RT-Real Time PCR. MG: noradrenalin release determination. HL: discussion and manuscript preparation. AP: participated in ovarian morphology analysis, noradrenalin release determination, and contributed to the development, design, coordination of the research, manuscript preparation and was responsible for the grant. All authors read and approved the final manuscript.
